# Bioenergetic and Metabolic Adaptation in Tumor Progression and Metastasis

**DOI:** 10.3389/fonc.2022.857686

**Published:** 2022-03-17

**Authors:** Patries M. Herst, Georgia M. Carson, David A. Eccles, Michael V. Berridge

**Affiliations:** ^1^ Department of Cell Biology, Malaghan Institute of Medical Research, Wellington, New Zealand; ^2^ Department of Radiation Therapy, University of Otago, Wellington, New Zealand

**Keywords:** bioenergetic flexibility, glycolysis-OXPHOS continuum, mito-nuclear gene expression, tumor progression and metastasis, tumor microenvironment (TME)

## Abstract

The ability of cancer cells to adjust their metabolism in response to environmental changes is a well-recognized hallmark of cancer. Diverse cancer and non-cancer cells within tumors compete for metabolic resources. Metabolic demands change frequently during tumor initiation, progression and metastasis, challenging our quest to better understand tumor biology and develop novel therapeutics. Vascularization, physical constraints, immune responses and genetic instability promote tumor evolution resulting in immune evasion, opportunities to breach basement membrane barriers and spread through the circulation and lymphatics. In addition, the unfolded protein response linked to the ubiquitin proteasome system is a key player in addressing stoichiometric imbalances between nuclear and mitochondrially-encoded protein subunits of respiratory complexes, and nuclear-encoded mitochondrial ribosomal protein subunits. While progressive genetic changes, some of which affect metabolic adaptability, contribute to tumorigenesis and metastasis through clonal expansion, epigenetic changes are also important and more dynamic in nature. Understanding the role of stromal and immune cells in the tumor microenvironment in remodeling cancer cell energy metabolism has become an increasingly important area of research. In this perspective, we discuss the adaptations made by cancer cells to balance mitochondrial and glycolytic energy metabolism. We discuss how hypoxia and nutrient limitations affect reductive and oxidative stress through changes in mitochondrial electron transport activity. We propose that integrated responses to cellular stress in cancer cells are central to metabolic flexibility in general and bioenergetic adaptability in particular and are paramount in tumor progression and metastasis.

## Introduction

An upsurge in interest in cancer cell metabolism and bioenergetics in the last decade has greatly increased our understanding of how developing cancer cells adapt to fluctuating changes in their microenvironment by adopting different metabolic strategies. Rather than aerobic glycolysis being an obligatory consequence of mitochondrial dysfunction as suggested by Warburg (1) almost 100 years ago, glycolysis in many tumors is upregulated without mitochondrial damage (2). Some authors now refer to this phenomenon as “selfish metabolic reprogramming” (3) or, the ability to attenuate OXPHOS if and when it suits to promote survival, proliferation, invasion and metastasis, an archetypal example of survival of the fittest.

Depending on the type of cancer cell, certain mutations and epigenetic changes can combine to severely restrict the respiratory capacity, and therefore mitochondrial energy production, in highly glycolytic cancer cells. Respiration-restricted cancer cells maximize carbon-conserving anabolic metabolism necessary for cell proliferation by optimizing glycolytic, glutaminergic and pentose phosphate pathways (4). In addition, highly glycolytic cells increase lactic acid dehydrogenase and plasma membrane electron transport activities to ameliorate reductive stress caused by build-up of NADH in cells with limited mitochondrial electron transport (5, 6). Cancer cells that are not respiration-restricted are advantaged by their ability to use both glycolytic and mitochondrial energy production strategies, depending on the availability of oxygen and nutrients. Under hypoxic conditions, many cancer cells quickly and effectively emulate the adaptations of their highly glycolytic competitors.

## The Glycolysis-OXPHOS Continuum

**Figure 1:**
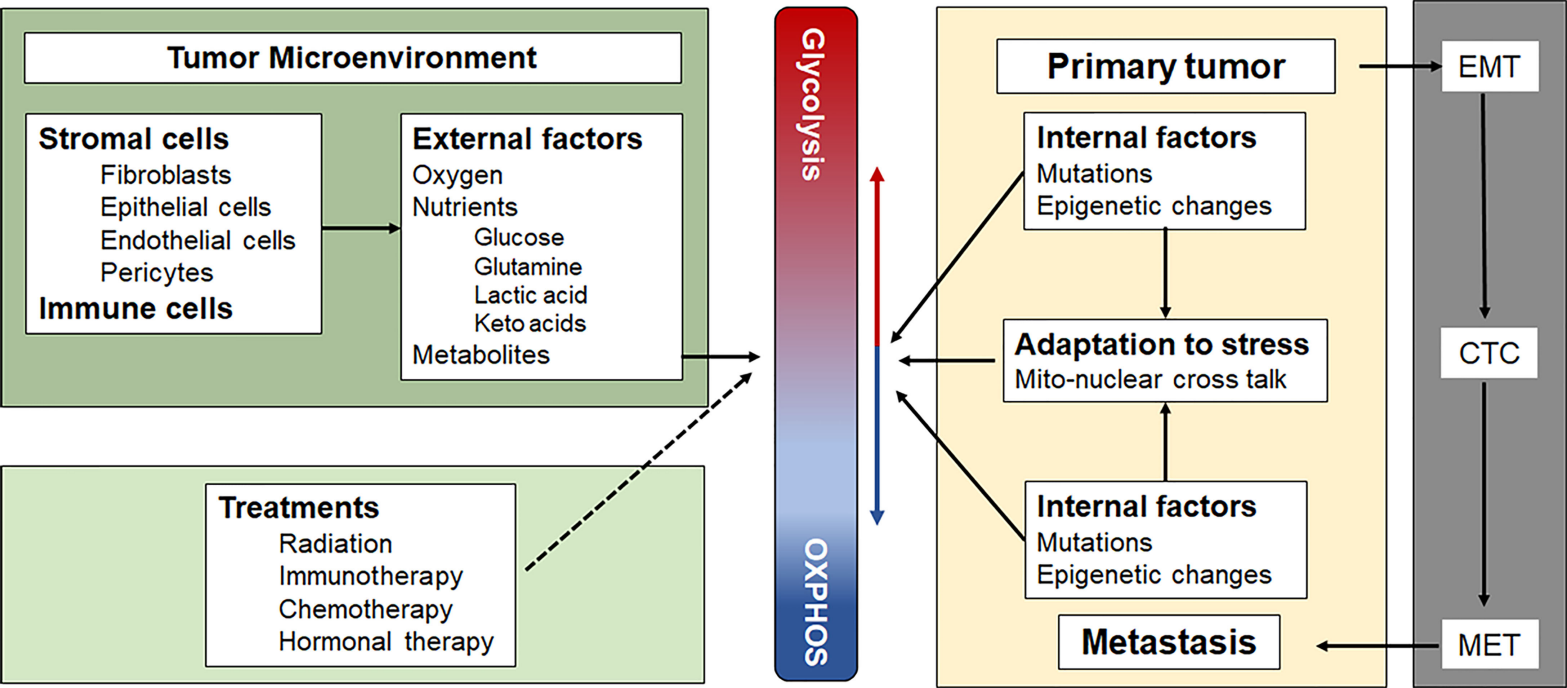
Factors that influence the position of a cancer cell on the Glycolysis-OXPHOS energy metabolism continuum. See text for a more detailed description. CTC, circulating tumor cells; EMT, epithelial to mesenchymal transition; MET, mesenchymal to epithelial transition.

Although the concept of a metabolic switch, which suggests OXPHOS can be turned on or off, is well established in the field, we (7) and others (8) have suggested that there is a continuum between an energy metabolism based largely on glycolysis and one based largely on OXPHOS. In the absence of external energy sources, C2C12 myocytes and HEK293 fibroblasts, were shown to obtain energy exclusively through OXPHOS by oxidizing endogenous substrates, such as glycogen (9). The addition of glucose resulted in a mixed energy metabolism with OXPHOS rates dropping 20% and glycolytic rates increasing 10-100 fold from near zero. Inhibiting OXPHOS increased glycolytic ATP production even further in some cell types, fully compensating for the loss of mitochondrially-generated ATP (9). Because of the greater efficiency of OXPHOS compared with substrate phosphorylation during glycolysis, cells only need to dedicate a very small part (4-6%) of their maximum OXPHOS capacity to contribute a large amount (40-50%) to their total energy budget (8). The ease with which cells can adjust the contributions of glycolysis and OXPHOS to their total energy budget has been called the “supply flexibility index” by Mookerjee et al. (8). These authors also present an elegant way to calculate the exact contributions from glycolysis and OXPHOS to the total energy budget based on raw oxygen consumption rate and extracellular acidification rate values measured with different substrates and inhibitors of glycolysis and OXPHOS, using the external flux Seahorse analyzer (8). The ability to finely adjust these contributions allows rapidly dividing cancer cells to respond quickly to changes in oxygen and nutrient levels. It further allows cells to balance the risks associated with high levels of reactive oxygen species (ROS) generated during stress and inefficient mitochondrial electron transport, notwithstanding the need for adequate ROS levels for essential signaling and mitogenic purposes under hypoxic conditions [reviewed in (10)]. At any point in time, each cancer cell in a tumor is somewhere on this continuum, depending on its genetic and epigenetic profile, its microenvironment and its location on the primary-metastatic tumor trajectory (**Figure 1**).

## Controlling the Glycolysis-OXPHOS Continuum

Master regulators of the Glycolysis-OXPHOS continuum are the hypoxia inducible factors (HIFs), transcription factors that respond to changes in oxygen levels [reviewed by (11)]. The heterodimer, HIF-1, is formed in hypoxic conditions when the oxygen-sensitive HIF-1α subunit is stabilized by coactivator proteins and translocated to the nucleus where it binds to the HIF-1β subunit. In normoxic conditions, the von Hippel–Lindau tumor suppressor protein (pVHL) binds to HIF-1α, resulting in ubiquination and destruction by the proteasome. The HIF-1 dimer binds to the hypoxia response element on DNA, resulting in transcription of genes that increase glycolytic activity through up-regulation of glucose transporters, glycolytic enzymes and lactate dehydrogenase (12). HIF-1 activation suppresses OXPHOS by inhibiting the flow of acetyl CoA into the TCA cycle through inhibition of pyruvate dehydrogenase. HIF-1 activation also increases the expression of vascular endothelial growth factor (angiogenesis) and erythropoietin (erythropoiesis), increasing oxygen and nutrient delivery [reviewed in (3, 11, 13). The importance of HIFs in adapting cellular metabolism in response to changes in oxygen levels was recognized by the 2019 Nobel Prize in Physiology or Medicine being awarded to Kaelin, Ratcliffe and Semenza, whose research groups were instrumental in elucidating HIF pathways (14).

## Genetic and Epigenetic Factors Affecting Energy Metabolism Strategies of Tumor Cells

The metabolic adaptation potential of individual cancer cells is determined by their genetic and epigenetic profiles. Nuclear and mitochondrial (mt)DNA mutations and epigenetically-driven transcriptional changes are increasingly recognized as factors that influence metabolic reprogramming, tumorigenesis, tumor progression, invasion and metastasis. At the regulatory level, any mutation or epigenetic change that increases HIF-1α stabilization will push a cancer cell towards increased glycolytic metabolism. For example, mutations that result in inability of pVHL to bind to HIF-1α will result in HIF-1 activation in the presence of oxygen, as was shown for pVHL mutations in renal carcinoma and hemangioblastoma (15). At the operational level, mutations or epigenetic changes that compromise OXPHOS, such as in nuclear genes that encode enzymes of the TCA cycle (16–18), the 79 protein subunits of the five respiratory complexes, the 77 mitochondrial ribosomal subunits, or genes that affect mtDNA copy number and complex assembly (19), will bias energy metabolism towards glycolysis.

Metabolic reprogramming in tumor progression and metastasis has been recently reviewed (20–22). Metabolic reprogramming can be seen in the requirement of a glycolytic phenotype, mediated by increased HIF-1α activity and PDK1 expression, to enable 4T1 breast cancer cells to metastasize to the liver, rather than to the bones or lungs (23). In metastatic melanoma xenograft models, increased expression of the monocarboxylate transporter, MCT1, increased metastatic potential (24). MCT1 is a bidirectional transporter which transports lactate out of highly glycolytic cells to maintain pH and an NAD+/NADH ratio conducive to sustaining high glycolytic rates *in vitro*. Interestingly, increased MCT1 expression in the melanoma xenograft models, led to an increase in lactate uptake which was directly related to an increase in metastatic potential (24, 25). Imported lactate is converted to pyruvate in the TCA cycle, generating NADH and a proton, which stimulates pentose phosphate pathway (PPP) activity by reducing both intracellular pH and the NAD+/NADH ratio. Lactate uptake through MCT1 as a preferred source of fuel over glucose *in vivo* has been described previously for human non-small cell lung cancer (NSCLC) (26). In metastatic lung cancer and pancreatic ductal adenocarcinoma, cumulative mutations in KRAS, the serine-threonine kinase, STK11, and the E3 ubiquitin ligase metastasis suppressor, KEAP1, establish an OXPHOS-driven phenotype, rendering these cells sensitive to OXPHOS inhibition, pyrimidine metabolism inhibitors and glutaminase inhibitors (20).

In addition to oxygen-dependent HIF regulation, oxygen-independent regulation of HIF-1α, so-called pseudohypoxia, can also activate HIF-1α through growth factor receptor mutations or activation of their respective mTOR signaling pathways. Examples include EGFR activation in lung adenocarcinoma, HER2 amplification in breast and gastric cancer, RAS mutations in lung and colorectal cancer, BRAF mutations in melanoma and mutations in the PI3K/AKT lipid kinase signaling pathways in various cancers (27).

Although mtDNA mutations are common across solid cancers, the contribution of mtDNA mutations to cancer progression and metastasis remains controversial, with a metabolic licensing model being proposed for some tumors (28, 29). Recent large scale molecular characterization of the mutational landscape of mitochondrial genomes in human cancers relative to matched control tissue identified truncations markedly enriched in kidney, colorectal and thyroid cancers. They further identified somatic mutation signatures with strand bias that were similar across tumor types suggestive of mtDNA polymerase (POLG) errors, and frequent nuclear transfers of mtDNA, some of which disrupt therapeutic target genes (30). In addition, multiplicity and heteroplasmy of mtDNA in cancer cells were reported as significant issues.

MicroRNAs have also been shown to contribute to metabolic regulation in cancer, for example by targeting hexokinase-2 to induce proliferative or quiescent responses in tumor spheroids (31–33). In addition, oncometabolites, such as D-2-hydroxyglutarate that accumulate in AML, gliomas, and colorectal cancers with IDH mutations, and inhibit dioxygenase enzymes in canonical metabolic pathways, are often involved in cancer metabolism by affecting epigenetic and post-translational processes and signal transduction [reviewed by (34)].

## Changes in Metabolic Reprogramming and the Glycolysis-OXPHOS Continuum Affecting Tumor Formation and Metastasis

Highly glycolytic cells express high levels of HIF-1α (11), produce fewer ROS, and have been associated with increased invasive and metastatic potential and poor patient outcomes (24, 25). However, even highly glycolytic cells retain some mitochondrial electron transport activity and a basic level of respiration. A complete absence of mitochondrial electron transport and respiration is incompatible with tumorigenesis *in vivo* as shown in metastatic mouse breast cancer (4T1) and melanoma (B16) cell lines lacking mtDNA (35). These ρ^0^ tumor cell lines are maintained in culture by supplementation with uridine and pyruvate. Uridine is required because mitochondrial electron transport generates ubiquinone, an essential cofactor for dihydroorotate dehydrogenase (Dhodh), the fourth enzyme in the *de novo* pyrimidine biosynthetic pathway (36). When injected into mice, tumor cell lines lacking mtDNA do not form tumors until they have acquired mitochondria, and therefore mtDNA, from adjacent stromal cells following a lag period of about 3 weeks. Knocking out the nuclear-encoded *Atp5b* gene and therefore mitochondrial ATP synthase activity in 4T1 and B16 cells, slowed but did not stop tumor growth demonstrating that OXPHOS itself is not a prerequisite for tumor formation. Thus, although ATP production is exclusively glycolytic in these cells, sufficient respiration remained to fuel pyrimidine production required for cell proliferation (36). These experiments functionally distinguish between the ATP-generating and respiratory functions of OXPHOS, and are supported by *Dhodh* knockout/knockin results that confirm the requirement of Dhodh for tumor growth (36). In other experiments, inserting the alternative oxidase gene, AOX, into 4T1 and B16 cells lacking mtDNA restored sufficient respiratory capacity to facilitate early tumor growth (36).

More recently, a similar conclusion regarding functional DHODH was reached using the human osteosarcoma cell line, 143B, using a four base-pair deletion in the *MT-CYTB* gene, an essential component of Complex III, and CRISPR-Cas9 gene editing to knock out the nuclear Complex III gene, *Uqcrq*, in the mouse lung cancer cell line, KP, and in a genetic model of T-cell ALL (37). In each model, tumor growth was abolished and mitochondrial ubiquinol oxidation and Dhodh activity were shown to be an absolute requirement for tumorigenesis.

Complex II is comprised of four nuclear-encoded subunits that link the TCA cycle with mitochondrial electron transport and respiration. It is an important alternative source of electrons contributing to mitochondrial electron transport alongside Complex I and DHODH, which can contribute about 10% to total mitochondrial oxygen consumption (36). Recently, Spinelli et al. showed that in human osteosarcoma 143B cells and in several normal tissues, fumarate can act as the terminal electron acceptor when oxygen is limiting, involving reverse electron flow through succinate dehydrogenase (SDH, Complex II) (38). This would have the effect of maintaining *de novo* pyrimidine biosynthesis by oxidizing ubiquinol, and thus ensuring continued Dhodh activity. The extent to which this mechanism supports coenzyme Q redox cycling and *de novo* pyrimidine production *in vivo* under conditions of varying oxygen availability is unclear. However, the potential for reverse electron flow through SDH to support cell proliferation in some tumors warrants further investigation.

The results described in the paragraphs above beg the question of whether or not mitochondrial ATP production is required for the maintenance of cancer stem cells, epithelial to mesenchymal transition (EMT), maintenance of circulating tumor cells, mesenchymal to epithelial transition (MET) and the establishment of metastases in distant organs. In this context, OXPHOS includes not only mitochondrial electron transport and ATP synthesis, but also coenzyme Q redox cycling, ROS production and proton pumping, linked processes that are evolutionarily conserved in higher eukaryotic organisms.

## The Mito-Nuclear Crosstalk Dilemma in Cancer Cell Energy Metabolism

**Figure 2:**
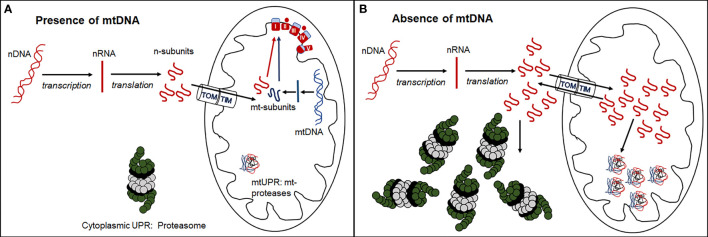
Formation of functional respiratory complexes in the presence **(A)** and absence **(B)** of mtDNA. **(A)** In the presence of adequate mtDNA transcription and translation, the nuclear- encoded subunits enter the mitochondria through the outer (TOM) and inner (TIM) mitochondrial membrane transporters and combine stoichiometrically with mitochondrially-encoded subunits to form functional respiratory complexes. **(B)** In the absence of mtDNA, no mitochondrially-encoded subunits are synthesized, and nuclear-encoded subunits are either directly degraded by the cytosolic UPR through the proteasome or enter mitochondria and are degraded by the mitochondrial UPR via mitochondrial proteases. Some of these subunits leave the mitochondria again through reverse transport through TOM and TIM and are degraded by the cytosolic proteasome.

The existence of tumor cell lines without mtDNA raises questions about mito-nuclear crosstalk in cells with severely compromised respiratory function. Mitochondrial respiratory complexes I, III, IV and V, are comprised of both mitochondrial and nuclear-encoded protein subunits (13 and 79 respectively), while mitochondrial ribosomes comprise 2 mitochondrially-encoded ribosomal RNAs and 77 nuclear-encoded subunits. In the absence of mtDNA, nuclear transcripts that contribute to these mitochondrial respiratory and ribosomal complexes continue to be synthesized in excess of need (39). Under normal physiological conditions, the balance between nuclear- and mitochondrially-encoded subunits of respiratory complexes is finely tuned (40–45). In many cancers, mitochondrial respiratory gene expression is suppressed (46), but it is currently unclear if an imbalance between mitochondrial and nuclear gene expression and protein synthesis may invoke cytosolic and mitochondrial unfolded protein stress responses (**Figure 2**) that have the potential to become novel targets in cancer treatment.

Differential gene expression analysis of 4T1 breast cancer cell lines with and without mtDNA showed that the nuclear gene encoding the immunoproteasome subunit, Psmb8, was poorly expressed in the absence of mtDNA, and that expression was restored in a cell line derived from a primary tumor that had acquired mitochondria/mtDNA (39). These results suggest that in cells with compromised respiration resulting from low mtDNA copy number, cytosolic immunoproteasome remodeling may remove excess nuclear-encoded respiratory and ribosomal subunits as part of the cytoplasmic unfolded protein response (UPR) (**Figure 2**).

Analysis of cell lines with partially compromised respiratory function linked to mtDNA copy number where differential expression of *Psmb8* may be intermediate between cell lines with and without mtDNA, may add to our understanding of mechanisms relating to imbalances in mitochondrial and nuclear-encoded respiratory subunit complexes. In addition to cytosolic proteosome activity, excess nuclear-encoded respiratory and mitochondrial ribosomal protein subunits, tagged with mitochondrial import signals, may be recognized by the mitochondrial UPR. When the mitochondrial UPR is overwhelmed (**Figure 2**), cellular stress and mitophagy may result. Exploration of the expression thresholds of these nuclear-encoded respiratory and ribosomal polypeptides may reveal targetable vulnerabilities of cancer cells biased towards proliferation, glycolytic metabolism and metastatic progression.

Mitochondrial DNA copy number is depleted in many solid tumors (47), while expression of different protein-encoding genes in mtDNA and even within a particular respiratory complex can vary by more than two orders of magnitude, with genes encoding subunits of Complex I *(Ndu1)* and V *(Atp6 and Atp8)* being the most highly expressed in 4T1 cells (38). In contrast to individual respiratory complexes where protein synthesis of mitochondrial and nuclear-encoded subunit genes are in general closely correlated across primary cells from different tissues and tumor cell lines (40–45), mitochondrial and cytosolic mRNA abundance for subunits of the respiratory complexes are less well matched (44). What is currently not known is if tumor cells with mtDNA but with low levels of respiration (e.g. hypoxia-adapted cells) also exhibit dysregulated expression of mitochondrial and nuclear genes encoding subunits of the respiratory complexes and mitochondrial ribosomes. Also unclear are the roles of nuclear-encoded respiratory complex assembly factors, mitochondrial and nuclear genetic and epigenetic processes, as well as intermediate metabolites and onco-metabolites, in maintaining the correct balance of mitochondrial respiratory complex subunits encoded by nuclear (n)DNA and mtDNA.

## The Primary Cancer-Metastatic Trajectory Affects Energy Metabolism Strategies

Although non-cancerous cells have limited plasticity with respect to their energy metabolism strategies, they tend to follow similar trends to cancer cells. Thus, rapidly dividing normal cells, activated immune cells, non-quiescent pluripotent and embryonic stem cells, progenitor cells, and myoblasts exhibit characteristics of aerobic glycolysis. As discussed earlier, this metabolic strategy supports anabolic metabolism, using carbon for building macromolecules rather than squandering it as carbon dioxide, a waste product of respiration.

Recent results involving rapidly dividing tumor cell lines suggest that base level respiration of about 10% of normal oxygen consumption is required to support cell proliferation (36). Another recent study identified fumarate as an alternative electron sink for maintaining ubiquinone levels essential for DHODH activity, *de novo* pyrimidine biosynthesis and nucleic acid production (38). Ideally, these strategies would be expected to minimize ROS production, and consequently reduce damage to mtDNA proximal to CI and CIII, where most superoxide radicals are generated. Where mitochondria are localized close to the nucleus, and where antioxidant and DNA repair mechanisms are overwhelmed, mutational nDNA damage would likely result. In contrast, non-dividing differentiated cells, such as muscle, brain and functionally differentiated cells in tissues, together comprising the majority of somatic cells in the body, rely on OXPHOS because of their high energy demands, and are less likely to be disadvantaged by high ROS levels. With either functionally differentiated non-dividing cells, or rapidly proliferating cells in tumors or non-cancer cells, cellular stress, senescence, aging or cellular damage will lead to inefficient mitochondrial electron transport, electron leakage and ROS production resulting in DNA damage, lipid peroxidation and protein oxidation.

Although initially thought to be highly glycolytic, stem cells and cancer stem cells, that can be in either a quiescent (dormant) or self-renewing state, are now known to exhibit significant levels of OXPHOS (48–51). However, efficient mitochondrial electron transport, and slow or intermittent self-renewal undoubtedly allows time for repair of cellular damage and maintenance of antioxidant defense mechanisms, which include mitochondrial and cytosolic enzymes such as superoxide dismutases, glutathione peroxidase and catalase.

## The Complex Role of the Tumor Microenvironment in Regulating Tumor Cell Energy Metabolism

The tumor landscape is diverse, with its multitude of cancer cells with different adaptive potential. In addition to highly proliferative cells and non-proliferating cells at different stages of differentiation, tumors also contain slowly self-renewing cancer stem cells or stem-like cells with limitless growth potential. Each of these cell types adapts their metabolic strategies to suit their own needs to thrive in a highly competitive tumor microenvironment, characterized by strongly fluctuating oxygen and nutrient levels caused by a highly compromised tumor microvasculature. The cellular components of the tumor microenvironment consist of tissue of origin cells, immune cells, vascular cells and stromal cells. Each of these cell types can either inhibit or promote tumor progression, invasion and metastasis, depending on their activation status, which in turn depends on the cytokine/chemokine environment produced through intercellular communication [reviewed in (52)]. The metabolic requirements of individual cancer cells further depend on their localization in either the early primary tumor, a large well established primary tumor, invading tumor cells, intravasating tumor cells, circulating tumor cells (CTCs), extravasating tumor cells or cells establishing themselves in a new metastatic niche, as successful metastasis is directly affected by the composition of the tumor microenvironment (48).

The metastatic process is punctuated by a number of challenges that cancer cells must overcome that require metabolic reprogramming. These challenges include: the endothelial to mesenchymal transition (EMT) which facilitates invasion, intravasation and survival as circulating tumor cells, and the mesenchymal to endothelial transition (MET) required for establishing micro metastases. Both hypoxia-mediated EMT and hypoxia-independent EMT pathways have been described (53). HIF-1α promotes EMT and invasion *via* activation of the TGF-ß-SMAD3 pathway in breast cancer patients (54), the Wnt/ß-catenin pathway in hepatocellular carcinoma (55) and prostate cancer (56), and hedgehog signaling in pancreatic cancer cells (57). In addition, there are a number of non-HIF-1α hypoxia-induced pathways that promote EMT, such as AMPK, PI3K-Akt-mTOR, NF-kB and MAPKs which have recently been reviewed (53). The oxidative environment in the blood stream is a major challenge for CTCs, with only 0.01% surviving to form metastases (20). Upregulation of the PPP to generate glutathione, a prominent intracellular antioxidant, is a feature of many metastatic cancers, including breast and lung cancer and melanoma (20). The establishment of metastases requires metabolic plasticity and flexibility to a new TME where nutrient availability is likely to be very different from that of the primary tumor TME (20, 21). Increased expression of enzymes involved in asparagine, proline and serine metabolism, increased uptake of pyruvate and lactate and acetate to provide alternative forms of energy and increased activity of the PPP have all shown to increase the metastatic ability of a number of cancers, such as breast and brain cancer and melanoma [recently reviewed in (22)].

Heterogeneity in the primary tumor can determine the site of metastasis; in melanoma, cells with an OXPHOS phenotype metastasize to the brain, whereas those that are more glycolytic metastasize to the lungs. Moreover, high MCT-1 expressing melanoma cells have a much higher metastatic potential than cells that express MCT-1 at a lower level (57).

## Summary and Future Perspectives

The bioenergetic and metabolic characteristics of cancer cells reflect many of the properties of normal self-renewing stem cells and their rapidly proliferating progenitors where commitment to differentiate, and differentiation *per se*, are markedly compromised or lost. Dedifferentiation is also known to contribute to the development of some cancers. In this context, differentiation induction as a cancer treatment strategy has been used successfully for several decades now, for example in the forced differentiation of acute promyelocytic leukemia by a derivative of vitamin A (53). Alterations in normal cell physiology that contribute to tumorigenesis, progression and metastasis are brought about by accumulating genetic and epigenetic changes that are associated with dynamic alterations in the tumor microenvironment. The complexity of these adaptive changes in tumor cells demands in-depth understanding of the individual changes involved, as well as system approaches that integrate hard-wired genetic and reversible epigenetic changes in individual cancers that affect nucleated cell types in the body that give rise to cancer.

Of particular interest in this context are the underlying changes in energy metabolism and metabolic reprogramming that are a common feature of tumor cell biology. This is particularly important because it involves contributions from both nuclear and mitochondrial genomes. These evolutionarily distinct genomes are interdependent and underpin the balance between glycolytic ATP production and ATP generated by oxidative phosphorylation in mitochondria. The simplistic dogma that rapidly proliferating cells, including tumor cells, progenitor cells and immune cells, use aerobic glycolysis while functionally-differentiated tissue cells depend on oxidative phosphorylation has become much more nuanced recently with the realization that stem cells, including cancer stem cells, exist in either quiescent or self-renewing states, and that rapidly-proliferating cells exhibit bioenergetic plasticity dictated by the availability of oxygen, glucose, glutamine and other nutrients. In reality, these cells seldom derive more than 50% of their ATP from glycolysis (1). In addition, genetic approaches have allowed oxidative phosphorylation to be separated into 1) respiration, essential for *de novo* pyrimidine and nucleic acid biosynthesis and therefore cell proliferation and tumor formation, and 2) mitochondrial ATP production *via* Complex V (ATP synthase) which is not essential for tumorigenesis (36).

Together, these results suggest multiple layers of regulation of respiratory complex production and function at the transcriptional and translational level of mitochondrial and nuclear respiratory complex gene and protein expression, as well as mitochondrial protein import and respiratory complex assembly, with rate-limiting steps in the assembly of each complex being a major factor (42, 44). Dynamic changes in the tumor microenvironment orchestrate epigenetic changes that in turn impact gene expression at each stage of tumor development, while intermediate metabolites are well-established as modulating factors in bioenergetic control which change dynamically over time (34, 54).

Better understanding of the bioenergetic and metabolic adaptations in tumorigenesis and metastasis will highlight treatment opportunities that target critical bioenergetic bottlenecks in primary tumor growth, CTC production, micro-metastases, and the metastatic progression that is responsible for most cancer mortality.

## Author Contributions

MB and PH: conceptual design of perspective article and joint primary authors and revision. GC and DE: contributions to authorship and revision. PH: designed **Figures 1** and **2**. All authors contributed to the article and approved the submitted version.

## Funding

MB, PH, GC, and DE are supported in part by the Health Research Council of New Zealand, Grant 20/506, and by the Malaghan Institute of Medical Research. PH was also supported by the Department of Radiation Therapy, University of Otago, Wellington.

## Conflict of Interest

The authors declare that the research was conducted in the absence of any commercial or financial relationships that could be construed as a potential conflict of interest.

## Publisher’s Note

All claims expressed in this article are solely those of the authors and do not necessarily represent those of their affiliated organizations, or those of the publisher, the editors and the reviewers. Any product that may be evaluated in this article, or claim that may be made by its manufacturer, is not guaranteed or endorsed by the publisher.

## References

[1] Warburg O. On Respiratory Impairment in Cancer Cells. Sci (1956) 124:269–70. doi: 10.1126/science.124.3215.269 13351639

[2] Koppenol WH Bounds PL Dang CV. Otto Warburg’s Contributions to Current Concepts of Cancer Metabolism. Nat Rev Cancer (2011) 11:325–37. doi: 10.1038/nrc3038 21508971

[3] Vaupel P Multhoff G. Revisiting the Warburg Effect: Historical Dogma Versus Current Understanding. J Physiol (2021) 599:1745–57. doi: 10.1113/JP278810 33347611

[4] DeBerardinis RJ Chandel NS. We Need to Talk About the Warburg Effect. Nat Metab (2020) 2:127–9. doi: 10.1038/s42255-020-0172-2 32694689

[5] Herst P Berridge M. Plasma Membrane Electron Transport: A New Target for Cancer Drug Development. Curr Mol Med (2006) 6:895–904. doi: 10.2174/156652406779010777 17168740

[6] Herst P Berridge M. Cell Surface Oxygen Consumption: A Major Contributor to Cellular Oxygen Consumption in Glycolytic Cancer Cell Lines. Biochim Biophys Acta - Bioenerg (2007) 1767:170–7. doi: 10.1016/j.bbabio.2006.11.018 17266920

[7] Herst PM Grasso C Berridge MV. Metabolic Reprogramming of Mitochondrial Respiration in Metastatic Cancer. Cancer Metastasis Rev (2018) 37:643–53. doi: 10.1007/s10555-018-9769-2 30448881

[8] Mookerjee SA Gerencser AA Nicholls DG Brand MD. Quantifying Intracellular Rates of Glycolytic and Oxidative ATP Production and Consumption Using Extracellular Flux Measurements. J Biol Chem (2017) 292:7189–207. doi: 10.1074/jbc.M116.774471 PMC5409486 28270511

[9] Mookerjee SA Nicholls DG Brand MD. Determining Maximum Glycolytic Capacity Using Extracellular Flux Measurements. PloS One (2016) 11:1–20. doi: 10.1371/journal.pone.0152016 PMC4816457 27031845

[10] Idelchik MdPS Begley U Begley TJ Melendez JA. Mitochondrial ROS Control of Cancer. Semin Cancer Biol (2017) 47:57–66. doi: 10.1016/j.semcancer.2017.04.005 28445781 PMC5653465

[11] Courtnay R Ngo D Malik N Ververis K Tortorella S Karagiannis T. Cancer Metabolism and the Warburg Effect: The Role of HIF-1 and PI3K. Mol Biol Rep (2015) 42:841–51. doi: 10.1007/s11033-015-3858-x 25689954

[12] Semenza GL. HIF-1: Upstream and Downstream of Cancer Metabolism. Curr Opin Genet Dev (2010) 20:51–6. doi: 10.1016/j.gde.2009.10.009 PMC2822127 19942427

[13] Xie Y Shi X Sheng K Han G Li W Zhao Q. PI3K/Akt Signaling Transduction Pathway, Erythropoiesis and Glycolysis in Hypoxia. Mol Med Rep (2019) 19:783–91. doi: 10.3892/mmr.2018.9713 PMC6323245 30535469

[14] Zhang Q Yan Q Yang H Wei W. Oxygen Sensing and Adaptability Won the 2019 Nobel Prize in Physiology or Medicine. Genes Dis (2019) 6:328–32. doi: 10.1016/j.gendis.2019.10.006 PMC6889041 31832511

[15] Semenza GL. HIF-1 Mediates the Warburg Effect in Clear Cell Renal Carcinoma. J Bioenerg Biomembr (2007) 39:231–4. doi: 10.1007/s10863-007-9081-2 17551816

[16] Linehan WM Rouault TA. Molecular Pathways: Fumarate Hydratase-Deficient Kidney Cancer - Targeting the Warburg Effect in Cancer. Clin Cancer Res (2013) 19:3345–52. doi: 10.1158/1078-0432.CCR-13-0304 PMC4447120 23633457

[17] Ratcliffe PJ. Fumarate Hydratase Deficiency and Cancer: Activation of Hypoxia Signaling? Cancer Cell (2007) 11:303–5. doi: 10.1016/j.ccr.2007.03.015 17418405

[18] Rizwan M Rasheed HA Tarjan G. Succinate Dehydrogenase Complex: An Updated Review. Arch Pathol Lab Med (2018) 142:1564–70. doi: 10.5858/arpa.2017-0285-RS 30289269

[19] Herst P Rowe M Carson G Berridge M. Functional Mitochondria in Health and Disease. Front Endocrinol (Lausanne) (2017) 8:e296. doi: 10.3389/fendo.2017.00296 PMC5675848 29163365

[20] Faubert B Solmonson A DeBerardinis RJ. Metabolic Reprogramming and Cancer Progression. Science (2020) 368:152. doi: 10.1126/science.aaw5473 PMC7227780 32273439

[21] Bergers G Fendt SM. The Metabolism of Cancer Cells During Metastasis. Nat Rev Cancer (2021) 21:162–80. doi: 10.1038/s41568-020-00320-2 PMC8733955 33462499

[22] Ohshima K Morii E. Metabolic Reprogramming of Cancer Cells During Tumor Progression and Metastasis. Metabolites (2021) 11:1–23. doi: 10.3390/metabo11010028 PMC7824065 33401771

[23] Dupuy F Tabariès S Andrzejewski S Dong Z Blagih J Annis MG. PDK1-Dependent Metabolic Reprogramming Dictates Metastatic Potential in Breast Cancer. Cell Metab (2015) 22:577–89. doi: 10.1016/j.cmet.2015.08.007 26365179

[24] Tasdogan A Faubert B Ramesh V Ubellacker JM Shen B Solmonson A. Metabolic Heterogeneity Confers Differences in Melanoma Metastatic Potential. Nature (2020) 577:115–20. doi: 10.1038/s41586-019-1847-2 PMC6930341 31853067

[25] Fischer GM Jalali A Kircher DA Lee WC McQuade JL Haydu LE. Molecular Profiling Reveals Unique Immune and Metabolic Features of Melanoma Brain Metastases. Cancer Discovery (2019) 9(5):628–45. doi: 10.1158/2159-8290.CD-18-1489 PMC6497554 30787016

[26] Faubert B Li KY Cai L Hensley CT Kim J Zacharias G. Lactate Metabolism in Human Lung Tumors. Cell (2017) 171:358–71. doi: 10.1016/j.cell.2017.09.019 PMC5684706 28985563

[27] Sebestyén A Kopper L Dankó T Tímár J. Hypoxia Signaling in Cancer: From Basics to Clinical Practice. Pathol Oncol Res (2021) 27:1–15. doi: 10.3389/pore.2021.1609802 PMC8262153 34257622

[28] Garcia-Heredia J Carnero A. Decoding Warburg’s Hypothesis: Tumor-Related Mutations in the Mitochondrial Respiratory Chain. Oncotarget (2015) 6:41582–99. doi: 10.18632/oncotarget.6057 PMC4747175 26462158

[29] Gammage PA Frezza C. Mitochondrial DNA: The Overlooked Oncogenome? BMC Biol (2019) 17:1–10. doi: 10.1186/s12915-019-0668-y 31286943 PMC6615100

[30] Yuan Y Ju YS Kim Y Li J Wang Y Yoon CJ. Comprehensive Molecular Characterization of Mitochondrial Genomes in Human Cancers. Nat Genet (2020) 52:342–52. doi: 10.1038/s41588-019-0557-x PMC7058535 32024997

[31] Subramaniam S Jeet V Clements JA Gunter JH Batra J. Emergence of microRNAs as Key Players in Cancer Cell Metabolism. Clin Chem (2019) 65:1090–101. doi: 10.1373/clinchem.2018.299651 31101638

[32] Muciño-Olmos EA Vázquez-Jiménez A López-Esparza DE Maldonado V Valverde M Resendis-Antonio O. MicroRNAs Regulate Metabolic Phenotypes During Multicellular Tumor Spheroids Progression. Front Oncol (2020) 10:1–14. doi: 10.3389/fonc.2020.582396 33425736 PMC7793838

[33] Pinweha P Rattanapornsompong K Charoensawan V Jitrapakdee S. MicroRNAs and Oncogenic Transcriptional Regulatory Networks Controlling Metabolic Reprogramming in Cancers. Comput Struct Biotechnol J (2016) 14:223–33. doi: 10.1016/j.csbj.2016.05.005 PMC4915959 27358718

[34] Huang S Wang Z Zhao L. The Crucial Roles of Intermediate Metabolites in Cancer. Cancer Manag Res (2021) 13:6291–307. doi: 10.2147/CMAR.S321433 PMC8364365 34408491

[35] Tan A Baty J Dong L Bezawork-Geleta A Endaya B Goodwin J. Mitochondrial Genome Acquisition Restores Respiratory Function and Tumorigenic Potential of Cancer Cells Without Mitochondrial DNA. Cell Metab (2015) 21:81–94. doi: 10.1016/j.cmet.2014.12.003 25565207

[36] Bajzikova M Kovarova J Coelho AR Boukalova S Oh S Rohlenova K. Reactivation of Dihydroorotate Dehydrogenase-Driven Pyrimidine Biosynthesis Restores Tumor Growth of Respiration- Deficient Cancer Cells. Cell Metab (2019) 29:399–416. doi: 10.1016/j.cmet.2018.10.014 30449682 PMC7484595

[37] Martínez-Reyes I Cardona LR Kong H Vasan K McElroy GS Werner M. Mitochondrial Ubiquinol Oxidation is Necessary for Tumour Growth. Nature (2020) 585:288–92. doi: 10.1038/s41586-020-2475-6 PMC7486261 32641834

[38] Spinelli JB Rosen PC Sprenger H-G Puszynska AM Mann JL Roessler JM. Fumarate is a Terminal Electron Acceptor in the Mammalian Electron Transport Chain. Science (2021) 374:1227–37. doi: 10.1126/science.abi7495 PMC8803114 34855504

[39] Grasso C Eccles DA Boukalova S Fabre MS Dawson RH Neuzil J. Mitochondrial DNA Affects the Expression of Nuclear Genes Involved in Immune and Stress Responses in a Breast Cancer Model. Front Physiol (2020) 11:1–12. doi: 10.3389/fphys.2020.543962 33329014 PMC7732479

[40] Scarpulla RC. Transcriptional Paradigms in Mammalian Mitochondrial Biogenesis and Function. Physiol Rev (2008) 88:611–38. doi: 10.1152/physrev.00025.2007 18391175

[41] Couvillion MT Soto IC Shipkovenska G Churchman LS. Synchronized Mitochondrial and Cytosolic Translation Programs. Nature (2016) 533:499–503. doi: 10.1038/nature18015 27225121 PMC4964289

[42] Tang JX Thompson K Taylor RW Oláhová M. Mitochondrial OXPHOS Biogenesis: Co-Regulation of Protein Synthesis, Import, and Assembly Pathways. Int J Mol Sci (2020) 21:1–32. doi: 10.3390/ijms21113820 PMC7312649 32481479

[43] Wiese M Bannister AJ. Two Genomes, One Cell: Mitochondrial-Nuclear Coordination *via* Epigenetic Pathways. Mol Metab (2020) 38:100942. doi: 10.1016/j.molmet.2020.01.006 32217072 PMC7300384

[44] Soto I Couvillion M Mcshane E Hansen KG Moran JC Barrientos A. Balanced Mitochondrial and Cytosolic Translatomes Underlie the Biogenesis of Human Respiratory Complexes. bioRxiv (2021):2021.05.31.446345. doi: 10.1101/2021.05.31.446345 PMC9361522 35945592

[45] Geldon S Fernández-Vizarra E Tokatlidis K. Redox-Mediated Regulation of Mitochondrial Biogenesis, Dynamics, and Respiratory Chain Assembly in Yeast and Human Cells. Front Cell Dev Biol (2021) 9:1–24. doi: 10.3389/fcell.2021.720656 PMC8452992 34557489

[46] Reznik ED Wang Q La K Schultz N Sander C. Mitochondrial Respiratory Gene Expression is Suppressed in Many Cancers. Elife (2017) 6:1–16. doi: 10.7554/eLife.21592 PMC5243113 28099114

[47] Reznik E Miller ML Şenbabaoğlu Y Riaz N Sarungbam J Tickoo SK. Mitochondrial DNA Copy Number Variation Across Human Cancers. Elife (2016) 5:1–20. doi: 10.7554/eLife.10769 PMC4775221 26901439

[48] Corbet C. Stem Cell Metabolism in Cancer and Healthy Tissues: Pyruvate in the Limelight. Front Pharmacol (2018) 8:1–7. doi: 10.3389/fphar.2017.00958 PMC5777397 29403375

[49] Coller HA. The Paradox of Metabolism in Quiescent Stem Cells. FEBS Lett (2019) 593:2817–39. doi: 10.1002/1873-3468.13608 PMC7034665 31531979

[50] Tsogtbaatar E Landin C Minter-Dykhouse K Folmes CDL. Energy Metabolism Regulates Stem Cell Pluripotency. Front Cell Dev Biol (2020) 8:1–16. doi: 10.3389/fcell.2020.00087 32181250 PMC7059177

[51] Bonnay F Veloso A Steinmann V Köcher T Abdusselamoglu MD Bajaj S. Oxidative Metabolism Drives Immortalization of Neural Stem Cells During Tumorigenesis. Cell (2020) 182:1490–1507.e19.10.1016/j.cell.2020.07.039 32916131 10.1016/j.cell.2020.07.039

[52] Herst P Dawson R Berridge M. Intercellular Communication in Tumor Biology: A Role for Mitochondrial Transfer. Front Oncol (2018) 8:e344. doi: 10.3389/fonc.2018.00344 PMC6121133 30211122

[53] Noguera NI Catalano G Banella C Divona M Faraoni I Ottone T. Acute Promyelocytic Leukemia: Update on the Mechanisms of Leukemogenesis, Resistance and on Innovative Treatment Strategies. Cancers (Basel) (2019) 11:1–21. doi: 10.20944/preprints201910.0159.v1 PMC6826966 31635329

[54] Fendt SM Frezza C Erez A. Targeting Metabolic Plasticity and Flexibility Dynamics for Cancer Therapy. Cancer Discovery (2020) 10:1797–807. doi: 10.1158/2159-8290.CD-20-0844 PMC7710573 33139243

[55] Zhang Q Bai X Chen W Ma T Hu Q Liang C. Wnt/β-Catenin Signaling Enhances Hypoxia-Induced Epithelial-Mesenchymal Transition in Hepatocellular Carcinoma *via* Crosstalk With HIF-1α Signaling. Carcinogenesis (2013) 34:962–73. doi: 10.1093/carcin/bgt027 23358852

[56] Jiang YG Luo Y He DL Li X Zhang LL Peng T. Role of Wnt/β-Catenin Signaling Pathway in Epithelial-Mesenchymal Transition of Human Prostate Cancer Induced by Hypoxia-Inducible Factor-1α. Int J Urol (2007) 14:1034–9. doi: 10.1111/j.1442-2042.2007.01866.x 17956532

[57] Lei J Ma J Ma Q Li X Liu H Xu Q. Hedgehog Signaling Regulates Hypoxia Induced Epithelial to Mesenchymal Transition and Invasion in Pancreatic Cancer Cells *via* a Ligand-Independent Manner. Mol Cancer (2013) 12:1–11. doi: 10.1186/1476-4598-12-66 23786654 PMC3699387

